# Experimental Determination of TDR Calibration Relationship for Pyroclastic Ashes of Campania (Italy)

**DOI:** 10.3390/s18113727

**Published:** 2018-11-01

**Authors:** Giovanna Capparelli, Gennaro Spolverino, Roberto Greco

**Affiliations:** 1 Dipartimento di Ingegneria Informatica, Modellistica, Elettronica e Sistemistica, Università della Calabria, 87036 Rende, Italy; giovanna.capparelli@unical.it; 2 Dipartimento di Ingegneria, Università della Campania ‘L. Vanvitelli’, 81031 Aversa, Italy; roberto.greco@unicampania.it

**Keywords:** TDR calibration relationship, pyroclastic soil, dielectric constant, soil bulk density

## Abstract

Time domain reflectometry (TDR) is one of the most widely used techniques for indirect determination of soil volumetric water content (*θ*). TDR measures the relative dielectric constant (*ε_r_*) which, in a three-phase system like the soil, depends on water, air, and solid matrix dielectric constants. Since dielectric constant of water is much larger than the other two, *ε_r_* of bulk soil mainly depends on water content. In many cases, the application of TDR requires a specific calibration of the relationship *θ*(*ε_r_*) to get quantitatively accurate estimates of soil water content. In fact, the relationship *θ*(*ε_r_*) is influenced by various soil properties, such as clay content, organic matter content, bulk density, and aggregation. Numerous studies have shown that pyroclastic soils often exhibit a peculiar dielectric behavior. In Campania (Southern Italy) wide mountainous areas are covered by layered pyroclastic deposits of ashes (loamy sands) and pumices (sandy gravels), often involved in the triggering of landslides induced by rainwater infiltration. Reliable field measurements of water content of such soils are therefore important for the assessment of landslide risk. Hence, in this paper, the *θ*(*ε_r_*) relationship has been experimentally determined on samples of typical pyroclastic soil of Campania, collected around Sarno, reconstituted with different porosities. The aim of the study is to identify specific calibration relationships for such soils based not only on empirical approaches. In this respect, a three-phase dielectric mixing model with a variable exponent is introduced, and the variable value of the exponent is related to the different dielectric properties of bond and free water within the soil pores.

## 1. Introduction

Based on the strong relationship between the apparent dielectric permittivity of soil, *ε_r_*, with its volumetric water content, *θ* [1,2], Time domain reflectometry (TDR) is one the most common indirect methods for estimating soil water content in the field. In addition to its limited invasiveness, the reason for such a widespread use relies on several non-debatable advantages, compared to other indirect techniques: TDR is cheap and safe, and does not require specific skills for its operation and maintenance. However, as for any indirect method, the accuracy of the measurement depends on the calibration relationship needed to estimate soil water content from the measured permittivity. In fact, although the first applications of TDR to water content measurements in sandy soils seemed to indicate that such a relationship could be considered independent of the soil type, i.e., the so-called universal calibration relationship proposed by Topp et al. [2], it was lately recognized that in many cases a specific calibration relationship should be determined to get quantitatively accurate estimates of water content of soils, e.g., Reference [3] and, more recently, of other porous materials e.g., References [4,5].

In the case of soils, the *θ*(*ε_r_*) relationship is affected by several properties, such as clay content [6], organic matter content [7,8,9], bulk density [9,10], and soil aggregation [11]. In this respect, some pyroclastic soils are characterized by extremely low bulk density owing to a metastable structure of micro-aggregates, and thus exhibit a peculiar dielectric behavior [12,13].

In Campania (Southern Italy) wide mountainous areas are covered by layered pyroclastic deposits of ashes (loamy sands) and pumices (sandy gravels), originated by several eruptions of the volcanic complexes of Somma-Vesuvius and Phegerean Fields that occurred during the last 40,000 years [14,15]. Textural and structural characteristics of the soils depend on the distance and the direction from the eruptive centers, as finer materials were carried further away by the winds blowing during the eruptions [16,17,18]. In particular, the ashes show fairly variable degrees of compaction, resulting in porosities ranging between 0.5 and 0.75 [19]. In the last decades, such soils have been involved in a number of catastrophic landslides, induced by soil wetting during rainwater infiltration. Hence, monitoring of water content in the field, usually carried out by means of TDR measurements, is of paramount importance for the management of landslide risk.

In order to gain more insight in the dielectric behavior of the pyroclastic soils of Campania, and how it affects the interpretation of TDR measurements, in this paper the *θ*(*ε_r_*) relationship has been experimentally determined on samples of pyroclastic soils collected in the area of Sarno, reconstituted with different porosities. The aim of the study is to identify specific calibration relationships for water content measurement by TDR, capable of taking into account the effects of variations of porosity and soil bulk density.

## 2. Principles of TDR

TDR is commonly used to measure soil moisture either in the laboratory or in the field, e.g., References [20,21,22,23,24,25]. As already pointed out, the volumetric water content (*θ*) is indirectly estimated through the measurement of the relative dielectric permittivity (*ε_r_*). The technique is in fact based on the measurement of the travel time of an electromagnetic wave moving back and forth along a metallic probe immersed in the soil. The speed of propagation of the wave (*V_p_*) depends on the bulk relative dielectric permittivity of the soil, in turn related to its water content, and can be determined as *V_p_* = (2*L*/*t*), *L* being probe length and *t* the travel time. From *V_p_*, the relative dielectric permittivity is readily obtained as *ε_r_* = (*c*/*V_p_*)^2^, in which *c* is the speed of light in the vacuum.

The dielectric permittivity of a heterogeneous medium depends on the spatial arrangement of the various constituent materials and on their dielectric permittivity. Wet soil is usually described as a three-phase medium (solid particles, water, and air). At ambient temperatures, the relative dielectric permittivity of free water is around 80, while the minerals constituting the solid skeleton of most soils have 3 ≤ *ε_r_* ≤ 7 and air behaves as a vacuum, with *ε_r_* = 1 [26]. Hence, the bulk relative dielectric permittivity of wet soil strongly depends on its water content [1], and it can be used as a proxy for its measurement by introducing a calibration relationship *θ*(*ε_r_*).

Several expressions of the calibration relationships for wet soils have been proposed, either empirical or with some theoretical basis. One of the first expressions is the third order polynomial proposed by Topp et al. [2], experimentally determined for soil samples with various textures:

(1) $$\;\theta =-5.3\times 10^{-2}+2.92\times 10^{-2}\varepsilon_{r}-5.5\times 10^{-4}\varepsilon_{r}^{2}+4.3\times 10^{-6}\varepsilon_{r}^{3}\;$$

Equation (1) fitted the experimental data of water content for all the tested soils, regardless of water salinity and temperature, with errors in the estimates of soil water content smaller than 0.013 [2]. Thanks to the good agreement with the experimental dielectric behavior of various soils, Equation (1) is often considered as a “universal” relationship, and it is used for TDR measurements without a soil-specific calibration. However, several studies indicate that the dielectric response to water content variations of many soils is quite different from Equation (1). Namely, soils rich of loamy and clayey fractions, with high organic matter content, and with low density due to micro-structural aggregation, such as some soils of volcanic origin, strongly depart from Equation (1); however, whenever a quantitatively accurate estimate of water content is required, a specific determination of the *θ*(*ε_r_*) relationship is recommended [27,28,29,30,31].

One of the first empirical expressions, considering the effects of soil bulk density, *ρ*, on the relationship between dielectric permittivity and soil water content was proposed by Malicki et al. [10].

(2) $$\;\sqrt{\varepsilon^{*}}=0.63+0.62\rho +8.18\;\theta \;$$

Another class of expressions of the TDR calibration relationship are the dielectric mixing models, which define soil permittivity as the power-law volumetric average of the dielectric constants, *ε_i_*, of the various constituents of the soil (typically three: solids, free water, and air).

(3) $$\varepsilon_{eff}^{\alpha }=\overset{N}{\underset{i=1}{\sum }}f_{i}\;\varepsilon_{i}^{\alpha }\;$$

In Equation (3), *N* represents the number of considered constituents, and *f_i_* are the volumetric fractions of each constituent, and the exponent *α* depends on the spatial arrangement of the mixture with the respect to the applied electromagnetic field [32]. For a heterogeneous layered medium, the exponent varies between −1, for an electromagnetic field orthogonal to the layers, and +1, for a parallel field [33]. 

Roth et al. [34] proposed a dielectric mixing model with three phases

(4) $$\varepsilon_{r}={\left[\theta \varepsilon_{w}^{\alpha }+\left(1-n\right)\varepsilon_{s}^{\alpha }+\left(n-\theta \right)\varepsilon_{a}^{\alpha }\right]}^{\frac{1}{\alpha }}\;$$

in which *ε_w_*, *ε_a_*, *ε_s_* are the relative permittivity of free water, air, and solids, respectively, and the exponent *α* was experimentally found close to 0.5, indicating an isotropic distribution of the three phases. The dielectric mixing models can be rewritten in a more compact form

(5) $$\;\theta =m\varepsilon_{r}^{\alpha }-b\;$$

in which *m* and *b* are, respectively:

(6) $$\;m=\frac{1}{\varepsilon_{w}^{\alpha }-\varepsilon_{a}^{\alpha }};\;b=\frac{\left(1-n\right)\varepsilon_{s}^{\alpha }+n\varepsilon_{a}^{\alpha }}{\varepsilon_{w}^{\alpha }-\varepsilon_{a}^{\alpha }}\;$$

Therefore, the constants *m* and *b* should be theoretically determined. However, in practical applications the parameters *α* and *ε_s_* cannot be considered known, and so Equation (5) is often used as an empirical relationship without theoretical basis, thus determining *m* and *b* by fitting experimental data. 

To better fit experimental values of bulk permittivity of wet soils, dielectric mixing models with four phases have been proposed. In fact, the dielectric constant of bound water is very different from free water, rather resembling that of ice, due to the electrical bonds limiting the freedom of polarization of water molecules [35]. According to the four-phase dielectric mixing model proposed by Dobson et al. [36], Equation (5) and parameter *m* in Equation (6) remain unchanged, while the expression of parameter *b* becomes:

(7) $$\;b=\frac{\left(1-n\right)\varepsilon_{s}^{\alpha }+n\varepsilon_{a}^{\alpha }+\theta_{bw}(\varepsilon_{bw}^{\alpha }-\varepsilon_{w}^{\alpha })}{\varepsilon_{w}^{\alpha }-\varepsilon_{a}^{\alpha }}\;$$

In Equation (7), *θ_bw_* and *ε_bw_* represent the volumetric fraction and the dielectric permittivity of bound water. 

The dielectric mixing model of Maxwell-De Loor [37] also considers four constituents of the soil:

(8) $$\;\varepsilon_{r}=\frac{3\varepsilon_{s}+2\left(\theta -\theta_{bw}\right)\left(\varepsilon_{w}-\varepsilon_{s}\right)+2\theta_{bw}\left(\varepsilon_{bw}-\varepsilon_{s}\right)+2\left(n-\theta \right)\left(\varepsilon_{a}-\varepsilon_{s}\right)}{3+\left(\theta -\theta_{bw}\right)\left(\varepsilon_{s}/\varepsilon_{w}-1\right)+\theta_{bw}\left(\varepsilon_{s}/\varepsilon_{bw}-1\right)+2\left(n-\theta \right)\left(\varepsilon_{s}/\varepsilon_{a}-1\right)}\;$$

Again, the parameters of Equations (6) and (7), as well as of Equation (8), may be obtained from theory, but more often they are identified by best fitting of experimental data.

## 3. Materials and Methods

**Figure 1:**
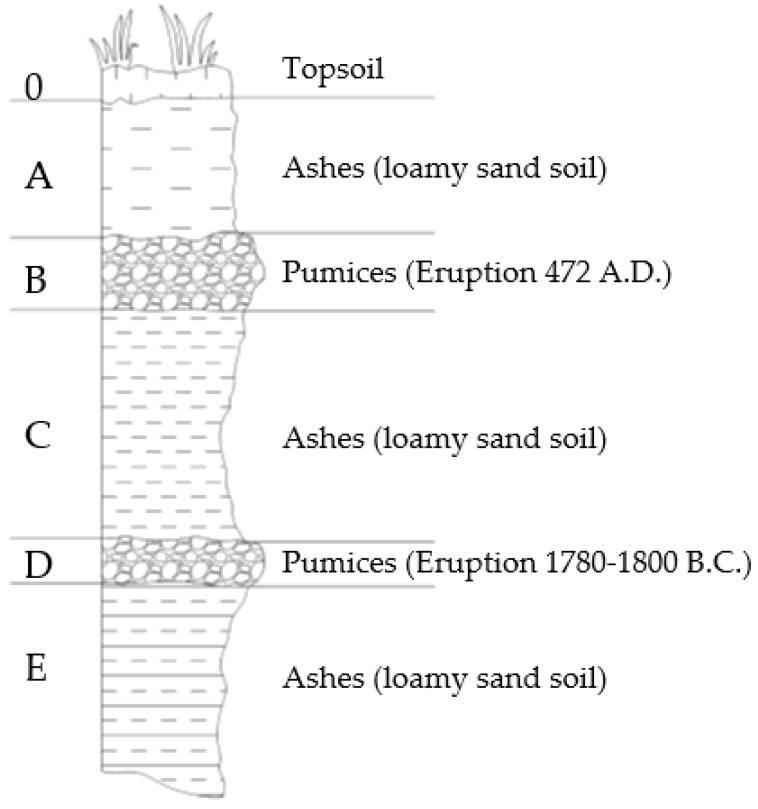
Sketch of the layered soil profile in the site of sample collection.

The tested soils are three volcanic ashes collected in the surroundings of Sarno (southern Italy), around 15 km NE of the crater of Vesuvius. In this area, complex layered air-fall pyroclastic soil deposits are found over a limestone bedrock (Figure 1). Generally, the soils of the deposits are cohesionless and consist of ashes (with texture ranging from sands, to loamy sands, and to loams) and pumices (from gravels to sands with gravel), e.g., Reference [38]. The tested ashes have been collected from layers A, C, and E of Figure 1, originated by different eruptions.

**Figure 2:**
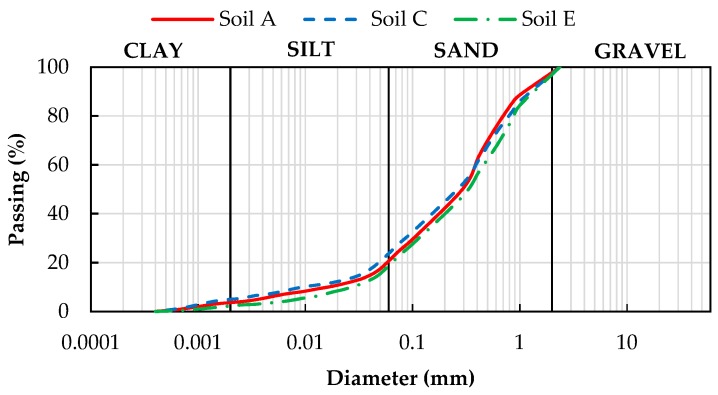
Particle size distribution curves of the three tested soils.

All the tested soils are loamy sands. The particle size distribution curves are shown in Figure 2. Table 1 reports the specific weight and the maximum diameter class of the solid particles, obtained with standard laboratory tests. More information about the physical properties of the pyroclastic soils of Campania can be found in Reference [39]. 

As clearly indicated by the curves of Figure 2, obtained with sieving and sedimentation tests, the three soils contain a small clay fraction, owing to the alteration having occurred before they were covered by deposits from more recent eruptions.

The experimental setup for TDR measurements consists of a TDR100 system (Campbell Scientific, Logan, UT, USA) connected to a three-rod metallic probe. The rods are 7.5 cm long, with diameter of 1.8 mm and spacing of 4 mm. The probe was vertically placed in the middle of a cylinder (diameter 10 cm, height 14 cm), inside of which soil samples of assigned porosity and water content were reconstituted. The method proposed by Heimovaara [40] has been used to measure the travel time of the signal from the acquired TDR waveforms. The total weight of dry soil needed to completely fill, after gentle compaction, the volume of the cylinder, *V_tot_*, to get a sample of assigned porosity, *n*, was obtained as:

(9) $$\;P_{s}=\left(1-n\right)\gamma_{s}V_{tot}\;$$

After oven drying for at least 24 h at the temperature of 105 °C, the smallest desired volumetric water content was obtained by adding to *P_s_* the weight of water, *P_w_*, given by:

(10)  θ=VWVtot=PwρwVtot 

The wet soil was gently mixed, to get a homogenous water content distribution, and was placed into the cylinder in five layers of equal weight. Then, the TDR probe was gently inserted into the soil. After at least two TDR measurements, the soil was removed from the cylinder, more water was added to obtain a higher water content, and the sample was placed again into the cylinder with the same procedure for mixing and reconstituting. The addition of water was repeated until the soil became a liquid mud, so that the reconstitution layer by layer of the sample was not possible anymore. For all the three investigated soils, the entire procedure was repeated with three different porosities (*n* = 0.50, 0.55, 0.60).

## 4. Results and Discussion

**Figure 3:**
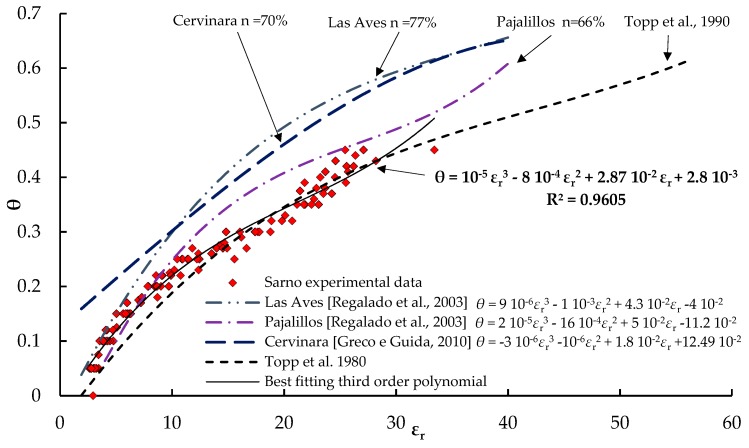
Experimental data of soil volumetric water content and relative dielectric permittivity compared with some empirical calibration curves from the literature. The best fitting third order polynomial of the experimental data is also plotted.

Table 2 reports all the measured (*θ*, *ε_r_*) couples acquired during the experiments for the three tested soils. The same data are plotted in Figure 3 and compared with the “universal” polynomial of Topp et al. [2], as well as with some calibration relationships of pyroclastic soils [12,13]. Although significant data spreading can be observed, mainly due to the differences of porosity, the experimental dots follow a distinct trend, which results closer to the equation of Topp et al. [2] than to the *θ*(*ε_r_*) relationships specific for other pyroclastic soils. However, it should be noted that both the relationships proposed by Regalado et al. [12] and by Greco et al. [13] refer to soils characterized by significantly higher porosity than the soils tested in this study, namely 0.77 (Las Aves) and 0.66 (Palajillos) for the two volcanic soils studied by Regalado et al. [12], and 0.70 for the pyroclastic ashes of Cervinara investigated by Greco et al. [13]. It looks clear that the soils from layers A, C, and E, although originated from different eruptions, show practically the same dielectric behavior. 

**Figure 4:**
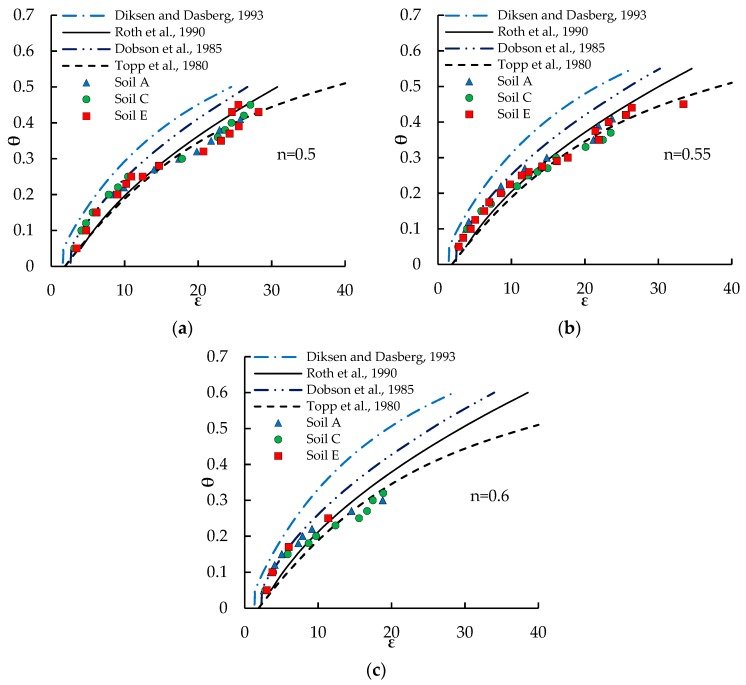
Experimental data for various porosities compared with calibration relationships from literature. (**a**) *n* = 0.5; (**b**) *n* = 0.55; (**c**) *n* = 0.6.

Aiming at investigating the effects of the changes in soil bulk density on the *θ*(*ε_r_*) relationship, the experimental data of samples with the same porosity have been grouped and are plotted in Figure 4. For each porosity, the trends described by the experimental data are compared with the calibration equations of Topp et al. [2], Dobson et al. [36], Roth et al. [34], and Maxwell-De Loor [37]. The parameters adopted for these models, reported in Table 3, have been chosen according to the literature for soils in which quartz and silica glass are the main minerals. In particular, for the estimation of *θ_bw_*, the formulation proposed by Dirksen and Dasberg [37] has been used.

None of the calibration relationships plotted in Figure 4 are able to fit the experimental data throughout the entire investigated ranges of water content and porosities. For all the investigated porosities, the expression of Topp et al. [2] fits well the experimental (*ε_r_*, *θ*) dots at high water contents, but underestimates the small water contents, especially for high porosities. Conversely, the four-phase model of Dobson et al. [36] performs well only at small water contents. The three-phase model of Roth et al. [34] falls in between the two previous models, so slightly underestimating *θ* for *ε_r_* < 10, and strongly overestimating it at high *ε_r_*. The RMSE of the various models adopted to fit the experimental data are reported in Table 4.

**Figure 5:**
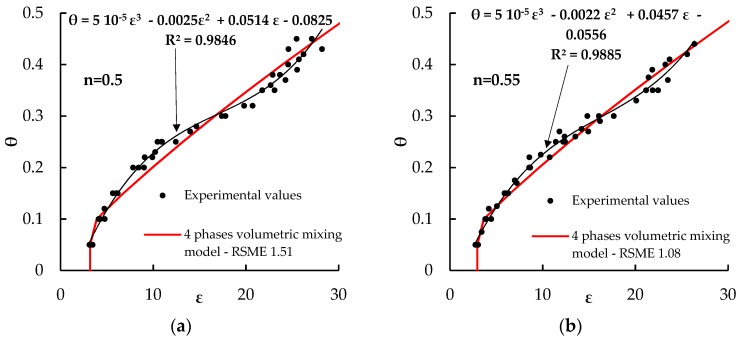

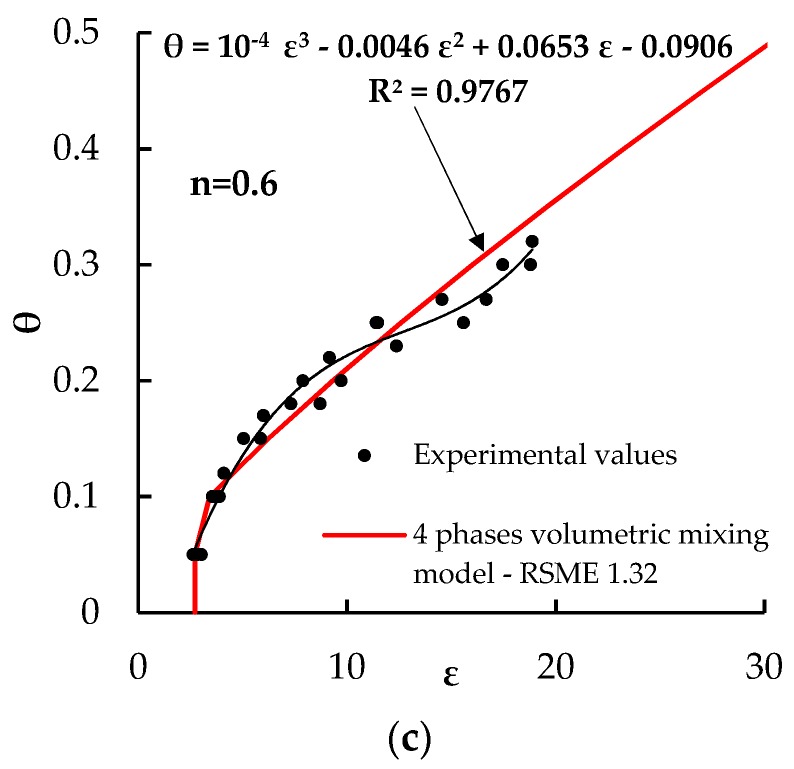
Fitting to the experimental data of the four-phase dielectric mixing model of Dasberg et al. [41], with calibrated parameters, for the various investigated porosities. (**a**) *n* = 0.5; (**b**) *n* = 0.55; (**c**) *n* = 0.6.

Aiming at identifying a better performing calibration equation, the parameters *ε_s_*, *ε_bw_*, *θ_bw_*, and *α* of the four-phase dielectric mixing model have been treated as calibration parameters, and they have been identified by minimizing the RMSE of the model compared to the experimental data. The obtained parameters are given in Table 5, and the corresponding best fitting curves are plotted in Figure 5. 

All the graphs of Figure 5 indicate that the four-phase dielectric mixing model, though satisfactorily fitting the data, is not capable of reproducing the inflection point in the *θ*(*ε_r_*) relationship shown by the experimental results of investigated soils, which has been highlighted by plotting in, Figure 5, the best fitting third order polynomials through the data (the same issue arises using a three-phase mixing model, as can be clearly seen in the plots of Figure 4).

**Figure 6:**
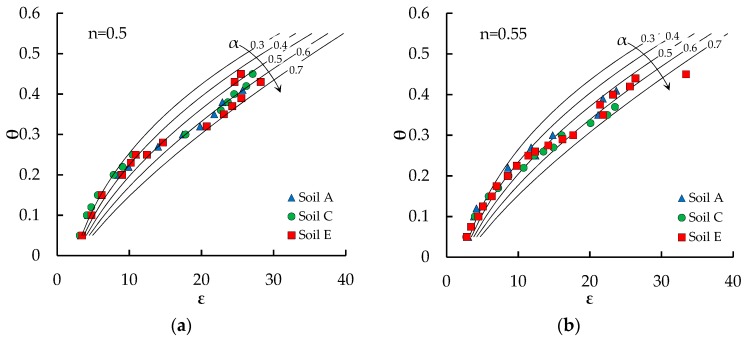

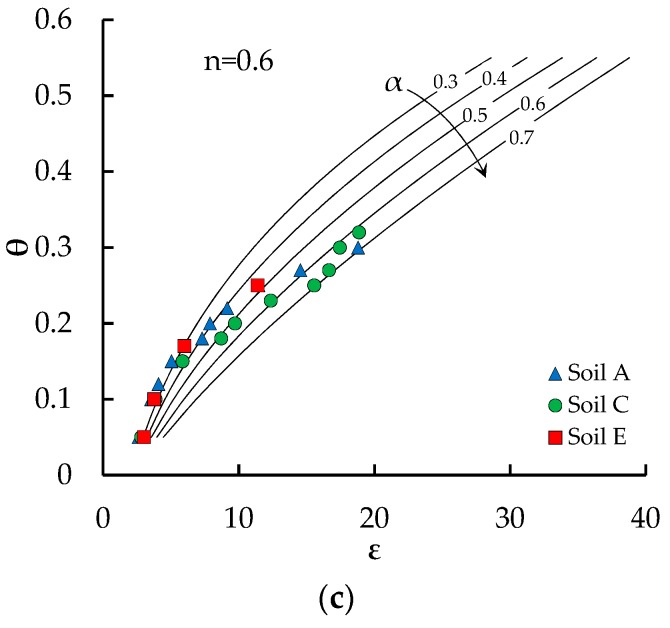
Experimental data at different porosities compared with the three-phase dielectric mixing model of Roth et al. [34] with various values of the exponent *α*. (**a**) *n* = 0.5; (**b**) *n* = 0.55; (**c**) *n* = 0.6.

Figure 6 shows that the peculiar trend of the *θ*(*ε_r_*) relationship of the investigated soils may be well interpreted by means of the three-phase dielectric mixing model of Roth et al. [34], with exponent *α* varying in the range 0.3 ≤ *α* ≤ 0.7.

In fact, Zakri et al. [33] pointed out that this parameter is influenced by soil structure, so that for structured soils it has been often considered as a fitting parameter of the model, e.g., References [9,12,30,42]. The pyroclastic soils of Campania, owing to the high specific surface of the particles and to the high content of allophanes in their mineral composition, usually exhibit a weak structure of micro-aggregates, e.g., References [38,43,44], which allows such soils to rest in extremely loose configurations, with porosities as high as 0.75, e.g., References [19,45], and it is reasonable to expect that the water assumes quite different spatial arrangements in the pores, moving from low water content (mainly bond water) to high water content (mainly free water).

**Figure 7:**
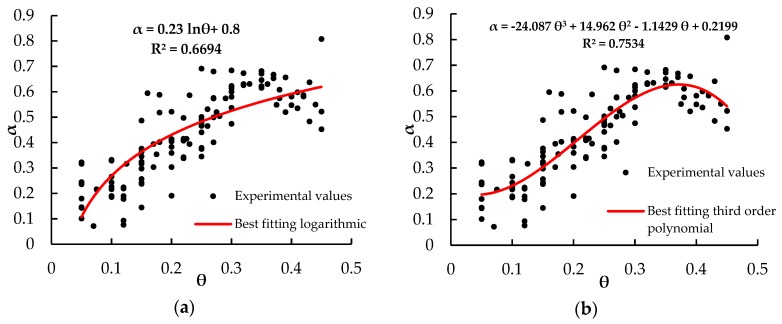
Values of the exponent *α* of the dielectric mixing model of Roth et al. [34] obtained, for all the experimental data, by forcing the model to give the same values of *ε_r_* as those provided by the TDR measurements. The red curves are fitting expressions of the *α*(*θ*) trend.

To gain more insight in the variability of *α*, Figure 7 shows the values obtained, for all the experimental data, by forcing the three-phase dielectric mixing model of Roth et al. [34] to give the same values of *ε_r_* as those provided by the TDR measurements, changing only *α* and keeping all the other parameters constant and equal to those reported in Table 3. The obtained values of the exponent *α*, clearly indicating a difference between small and high values of the water content, show an increasing trend of *α* up to *θ* slightly higher than 0.3 (i.e., the value at which the *θ*(*ε_r_*) experimental points show a trend change), and then slightly decreasing for higher values. The obtained values of *α* have been fitted with two expressions, also plotted in Figure 7.

**Figure 8:**
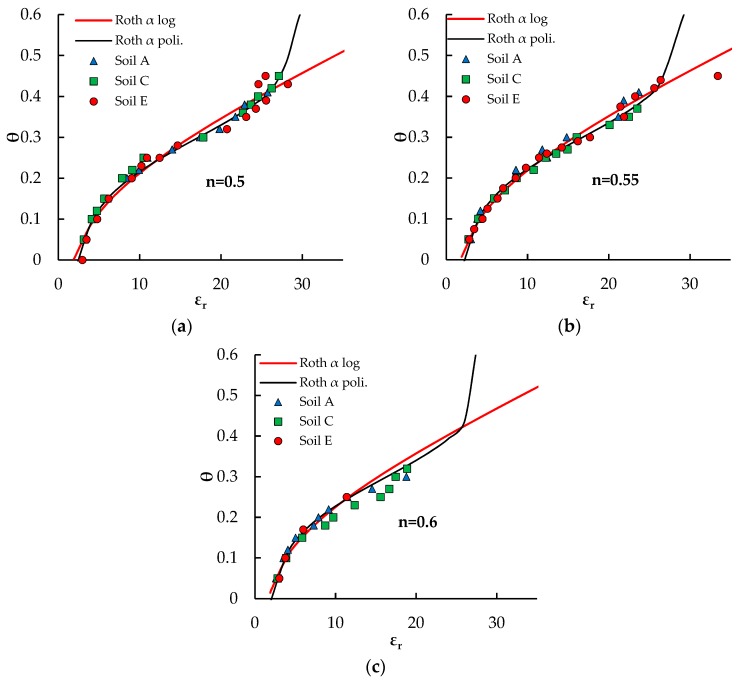
Experimental data compared with calibration curves obtained with the dielectric mixing model of Roth et al. [34] with variable exponent *α*. Two different expressions of the *α*(*θ*) relationships are represented.

The obtained best fitting expressions of the *α*(*θ*) relationships have been introduced in Equation (4) of the model of Roth et al. [34]. The resulting calibration curves are plotted in Figure 8 for the four investigated porosities.

Both of the obtained calibration curves fit very well the experimental points, but the polynomial *α*(*θ*) expression produces unrealistic values of water content for *ε_r_* > 27. Hence, the TDR calibration relationship of the pyroclastic soils of Sarno assumes the following expression:

(11) $$\begin{array}{cc} \varepsilon_{r}={\left[\theta \varepsilon_{w}^{\alpha }+\left(1-n\right)\varepsilon_{s}^{\alpha }+\left(n-\theta \right)\varepsilon_{a}^{\alpha }\right]}^{\frac{1}{\alpha }} & \mathrm{with}\;\alpha =0.23\ln\theta +0.8 \end{array}\;$$

As the logarithmic expression adopted for *α*(*θ*) assumes realistic values of the exponent even outside the investigated water content range, Equation (11) can be used for any water content from dry conditions to complete soil saturation.

## 5. Conclusions

The pyroclastic soils of Campania (Southern Italy) are characterized by extremely loose arrangements of particles, with porosities ranging between 0.5 and 0.75, and exhibit a peculiar dielectric response to moisture changes, which requires the identification of a specific calibration relationship linking the relative dielectric permittivity, *ε_r_*, to the volumetric water content, *θ*, for the measurement of soil water content with TDR.

In this paper, the *θ*(*ε_r_*) calibration relationship has been experimentally determined for three pyroclastic soils of Campania, reconstituted with various porosities. The investigated soils (sands with loam) were collected from different horizons of the layered profile of the area of Sarno (15 km North East of Naples).

Although originated by different eruptions of the volcanic complex of Somma-Vesuvius, occurring in a time interval of several thousands of years, the three investigated soils show the same dielectric response to water content changes when they are reconstituted with the same porosity. Even in absence of information about soil porosity, an empirical calibration equation has been obtained, which can be used with acceptable errors. 

The observed experimental trends have also been interpreted with several models of the calibration relationship proposed in literature, but none of the models satisfactorily fit the experimental data. At the lowest reconstituted porosity (*n* = 0.5), the experimental *θ*(*ε_r_*) trend results are close to the “universal” calibration relationship of Topp et al. [2], while it departs from it for increasing porosity. The departure from the relationship of Topp et al. [2] mainly affects the low water contents, suggesting that it may be related to the dielectric behavior of bound water, well known to be different from that of free water. Nevertheless, even a four-phase (solids, bound water, free water, and air) dielectric mixing model, specifically calibrated to best fit the experimental data, although performing better than the other tested models, does not successfully reproduce the observed trend. In dielectric mixing models, the exponent *α* of the volumetric average of the dielectric permittivity of the various phases account for their spatial arrangement and on how they interact with the electromagnetic field. As it can be expected that, in a structured soil, the spatial arrangement of bound water might be quite different from that of free water, a three-phase (solids, water, and air) dielectric mixing model with a variable exponent *α*(*θ*) is proposed to describe the calibration relationship *θ*(*ε_r_*), obtaining a very good fitting of the experimental data at all the investigated porosities.

Although the approach based on the different spatial distribution of bound water compared to free water can also be adopted for other pyroclastic soils, as they show similar properties, the obtained expression cannot be extended to other pyroclastic soils of Campania without a prior experimental verification. In fact, even if such soils have fairly similar properties, different grain size distributions and porosities could significantly affect their dielectric behavior, thus leading to different parameters in the logarithmic expression of *α*(*θ*).

## Figures and Tables

**Table 1:** Specific weight and maximum diameter class of the tested soils.

Sample	*γ_s_* (g/cm^3^)	d_max_ (mm)
A—Sarno	2.621	1.4 < d < 2.36
C—Sarno	2.542	1.4 < d < 2.36
E—Sarno	2.613	1.4 < d < 2.36

**Table 2:** Experimental data of soil volumetric water content and dielectric permittivity of the three investigated soils for the different tested porosities.

*n*	Soil A		Soil E		Soil C	
	*θ* (m^3^/m^3^)	*ε*	*θ* (m^3^/m^3^)	*ε*	*θ* (m^3^/m^3^)	*ε*
0.50	0.05	3.26	0.05	3.46	0.05	3.12
	0.10	4.26	0.10	4.76	0.10	4.11
	0.15	6.03	0.15	6.17	0.12	4.72
	0.20	8.42	0.20	9.01	0.15	5.64
	0.22	9.90	0.23	10.20	0.20	7.82
	0.25	10.95	0.25	10.89	0.22	9.07
	0.27	13.98	0.25	12.44	0.25	10.46
	0.30	17.40	0.28	14.66	0.30	17.80
	0.32	19.80	0.32	20.72	0.36	22.67
	0.35	21.76	0.35	23.09	0.38	23.66
	0.38	22.89	0.37	24.27	0.40	24.55
	0.41	25.72	0.39	25.53	0.42	26.21
			0.43	24.59	0.45	27.10
			0.43	28.21		
			0.45	25.46		
0.55	0.05	3.04	0.05	2.83	0.05	2.74
	0.10	3.80	0.08	3.43	0.10	3.93
	0.12	4.19	0.10	4.45	0.15	5.88
	0.15	6.05	0.13	5.07	0.17	7.21
	0.20	8.57	0.15	6.30	0.20	8.66
	0.22	8.57	0.18	6.99	0.22	10.76
	0.25	12.42	0.20	8.54	0.25	12.22
	0.27	11.81	0.23	9.80	0.26	13.53
	0.30	14.82	0.25	11.41	0.27	14.91
	0.35	21.16	0.26	12.36	0.30	16.06
	0.39	21.84	0.28	14.20	0.33	20.10
	0.41	23.69	0.29	16.18	0.35	22.46
			0.30	17.67	0.37	23.51
			0.35	21.87		
			0.38	21.42		
			0.40	23.24		
			0.42	25.59		
			0.44	26.37		
			0.45	33.42		
0.60	0.05	2.61	0.05	3.01	0.05	2.80
	0.10	3.53	0.10	3.77	0.10	3.87
	0.12	4.09	0.17	5.99	0.15	5.85
	0.15	5.04	0.25	11.38	0.18	8.70
	0.18	7.30			0.20	9.71
	0.20	7.88			0.23	12.36
	0.22	9.15			0.25	15.57
	0.25	11.46			0.27	16.66
	0.27	14.54			0.30	17.45
	0.30	18.79			0.32	18.87

**Table 3:** Parameters of theoretical calibration equations of Dobson et al. [36], Roth et al. [34], and Maxwell-De Loor [37].

*ε_s_*	*ε_w_*	*ε_a_*	*α*	*θ_bw_*	*ε_bw_*
5	78.5	1	0.5	0.054	3.2

**Table 4:** RMSE of the models adopted to fitting the experimental data.

*n*	Diksen and Dasberg, 1993	Dobson et al., 1985	Roth et al., 1990
0.5	6.07	3.90	2.08
0.55	6.22	3.67	1.97
0.6	5.13	3.14	1.92

**Table 5:** Best fitting parameters of the four-phase dielectric mixing model by Dobson et al. [36].

*ε_s_*	*ε_bw_*	*θ_bw_*	*α*
5.68	1.06	0.086	0.82
